# Photodegradation of Biohazardous Dye Brilliant Blue R Using Organometallic Silver Nanoparticles Synthesized through a Green Chemistry Method

**DOI:** 10.3390/biology10080784

**Published:** 2021-08-17

**Authors:** Agnieszka Sidorowicz, Tomasz Szymański, Jakub Dalibor Rybka

**Affiliations:** 1 Center for Advanced Technology, Adam Mickiewicz University, Uniwersytetu Poznańskiego 10 Street, 61-614 Poznan, Poland; sid.agnieszka@gmail.com (A.S.); tomszy1@amu.edu.pl (T.S.); 2 Faculty of Biology, Adam Mickiewicz University, Uniwersytetu Poznańskiego 6 Street, 61-614 Poznan, Poland; 3 Faculty of Chemistry, Adam Mickiewicz University, Uniwersytetu Poznańskiego 8 Street, 61-614 Poznan, Poland

**Keywords:** green chemistry, green synthesis, silver nanoparticles, photodegradation, dye degradation, *C. intybus*, organometallic silver nanoparticles

## Abstract

**Simple Summary:**

In the paper, we utilize silver nanoparticles as a catalyst in the degradation of a hazardous dye. The nanoparticles are formed from the simple silver salt by using only a plant extract from a commonly occurring herb. The plant extract contains compounds that can both reduce the silver salt and subsequently cap the surface of the as-prepared particles. There are many environmental advantages to using such an approach—nanoparticles are prepared by using simple green chemistry and the catalytic degradation of dye is carried out by sunlight energy. Such a method can be used as a very cheap, green method to neutralize hazardous substances in-house.

**Abstract:**

Nowadays, nanostructures having tremendous chemical and physical properties are gaining attention in the biomedical industry. However, when they are prepared through classical methods (physical and chemical), they are often non-biocompatible and toxic. Considering the mentioned factors, in this research, organometallic silver nanostructures (OMAgNs) have been prepared by the green chemistry method using the acetone, methanol, and methanol-hexane-based extracts of the medicinally important plant *Cichorium intybus*. Secondary metabolites from *C. intybus* can be used as an alternative to synthetic reagents at an industrial scale to manufacture biosafe and economical nanostructures with enhanced physicochemical parameters. Prepared nanostructures were characterized using SEM, XRD, FTIR, TGA, UV, and zeta potential measurement. SEM analysis revealed different shapes of OMAgNs, prepared with various extracts. XRD analysis showed the crystallinity of the nanostructures. FTIR spectroscopy helped to identify groups of compounds present in the extracts and used for the OMAgNs synthesis. Out of the three tested OMAgNs, those prepared with methanol extract were selected due to the highest obtained yield and stability (highest negative zeta potential) and were tested as a cost-efficient and active agent to photodegrade organic pollutant, Brilliant Blue R, using energy from sunlight. A decrease in UV-VIS absorbance confirmed the rapid degradation of the dye.

## 1. Introduction

Many medical or clinical laboratories deal with the problem of potentially biohazardous pollutants as a result of various chemicals used during tests. One of the most common procedures in the laboratory is staining, which enhances a contrast in samples at a microscopic level. While useful in scientific and medical studies, dyes can cause toxic interactions inside a living cell of human skin, causing harmful mutations [1]. Combined with a considerable amount of water during a series of washing, dyes can also be a source of water waste [2]. The solution to this problem is complex, as many types of organic pollutants need to be degraded.

To prevent water pollution, hazardous wastes after usage are disposed of into separate containers and sent to specialized water treatment facilities, where the waste undergoes processing. There are many physical, chemical, and biological treatment methods, such as carbon adsorption, neutralization, oxidation, membrane filtration, and biodegradation, often used in combination [3]. Each of these methods has its limitations in terms of cost, feasibility, efficiency, environmental impact, pre-treatment requirements, and formation of potentially toxic by-products. Despite the number of available treatment methods, only a few are commonly used by companies due to various economic and technological reasons. Hence, there is a need to develop new, cheaper, and more environmentally-friendly processes.

Green chemistry is an intensively researched concept, which aims at minimizing the environmental footprint, using more sustainable methods in chemical engineering. One of the approaches is to use plant extracts in the synthesis of nanoparticles. It is considered a more environmentally friendly method compared to chemical and physical methods. Additionally, as-prepared nanoparticles tend to be less toxic because of the lack of impurities after synthesis. Plant extracts are composed of enzymes and phytochemicals, which can act as reducing agents to metal salt and then as capping compounds, which prevent aggregation and are partly responsible for their properties [4]. One of the first green syntheses of metal nanoparticles was performed by Raveendran et al., in 2009, utilizing starch solution as a reducing agent for AgNO_3_ [5] and Begum et al., with Black Tea leaf extract [6]. In particular, organometallic silver nanoparticles (OMAgNs) have attracted scientists due to their catalytic [7], antimicrobial [8], antioxidant [9], and anticancer properties [10].

Since then, silver nanoparticles were prepared with various plant extracts, such as *Solanum tricobatum*, *Syzygium cumini*, *Centella asiatica*, *Citrus sinensis* [11], *Tribulus Terrestris* [12], *Alternanthera dentate* [13], and *Acorus calamus roots* [14]. Nanoparticles, due to their high surface area to volume ratio, can be used in many areas. Manimegalai et al. used silver nanoparticles to precipitate pesticides on a cellulose acetate membrane by adsorption and precipitation [15]. Fouda et al. used OMAgNs synthesized by using rice starch as a nanofertilizer for onion (*Allium cepa* L.) and showed highest crop yield using much lower concentration than in the commercially available fertilizers [16].

One of the plants used for the green synthesis is *Cichorium intybus*, a medicinally important herb belonging to the family *Asteraceae*, with significant distribution areas in Europe, Asia, Africa, and North America. The first-time leafy plant extract of *C. intybus* was used to synthesize silver nanoparticles (AgNPs). The Raman spectroscopy revealed the presence of ligands on AgNP, contributing to the reduction of silver ions. Antibacterial activity of synthesized products was tested against *Staphylococcus aureus*, *Escherichia coli*, and *Pseudomonas aeruginosa*, revealing its effectiveness at a picomolar concentration [17]. The extract was also tested for apoptotic properties in breast cancer cell lines [18] and the synthesis of copper [19] and gold nanoparticles [20].

Brilliant Blue R dye, also known as Coomasie, is widely used in laboratories for protein detection in sodium dodecyl sulfate-polyacrylamide (SDS-PAGE) gels, with a detection limit of a few nanograms of protein [21]. The procedure involves methanol and acetic acid, known as volatile compounds, further heated to shorten the staining process [22,23]. Methanol and acetic acid vapors have been documented as toxic, leading to many diseases [24,25]. Although the data concerning the hazardous nature of Brilliant Blue R dye is limited, in the official classification by the International Agency for Research on Cancer, it is placed in the 2B group as possibly carcinogenic to humans [26]. Exposure to the dye may irritate the skin, eyes, and respiratory system and cause adverse effects to the aquatic environment [27]. Even with concentration below the desired limits, the dye can impart color, posing a risk to the ecosystem [28]. In addition, Brilliant Blue R belongs to the group of the most difficult to remove reactive dyes. Hence, the development of degradation methods is needed to ensure a safe and clean environment.

Aqueous extracts of *Cirsium japonicum* and *Rosa brunonii* Lindl were used previously to prepare silver nanoparticles and use them as a photocatalysts to degrade dyes such as bromophenyl blue [29] and Congo red [30], respectively. In these papers, UV lamps were used as a photodegrading agent.

In this work, for the first time, we prepared three non-aqueous extracts of *C. intybus* leaves and used them as reducing and stabilizing agents to synthesize organometallic silver nanoparticles (OMAgNs). After the characterization of extracts and OMAgNs, we chose the most hydrophilic and abundant OMAgNs and tested their photocatalytic activity in reducing the Brilliant Blue R dye. To our knowledge, it is the first time that green synthesized OMAgNs prepared with *C. intybus* extracts have been used as a photocatalyst for rapid degradation of toxic dye using only sunlight (green energy).

## 2. Materials and Methods

### 2.1. Collection and Processing of Plant Material

Briefly, 180 g of *C. intybus* leaves and flowers were collected and washed with deionized H_2_O. For extract preparation, plant material was finely ground in a mortar with liquid nitrogen, then divided into 3 parts, 60 g each. Each prepared plant powder was added to flasks containing one of the following: 300 mL of acetone (Avantor™, Gliwice, Poland), 300 mL of 99.8% methanol (Basic, Avantor™, Gliwice, Poland), and 300 mL of hexane (HPLC Grade, Sigma Aldrich™, Burlington, MA, USA). The mixtures were transferred to a hot plate magnetic stirrer (MR Plug & Play Multi-Well Hei-Standard, Heidolph™, Schwabach, Germany) and agitated at a speed of 1000 rpm for 20 min under ambient conditions. In the next step, the mixtures were placed in an ultrasonic bath (Sonorex Digiplus Badelin™, Berlin, Germany) for the next 20 min. The flasks were then placed undisturbed under ambient conditions for 30 min, following filtration using Whatman No. 1 filter paper. In the next step, the extracts were dried under vacuum at 40 °C using a rotary evaporator (Hei-Vap, Heidolph™, Germany) until the volume was reduced to 90 mL. Obtained extracts were divided into three flasks: (a) 45 mL of acetone extract with 255 mL ddH_2_O; (b) 45 mL of methanol extract with 255 mL ddH_2_O; (c) 30 mL of methanol extract with 255 mL ddH_2_O. Finally, the mixtures were filtered using Whatman No. 1 filter paper.

### 2.2. Synthesis of OMAgNs through C. intybus Leaves and Flowers Extract

For the green synthesis of OMAgNs, 300 mL of *C. intybus* extracts were heated on a hot plate magnetic stirrer (MR Plug & Play Multi-Well Hei-Standard, Heidolph™) to 85 °C and stirred at ~1500 rpm for 15 min, then 5.1 g of silver nitrate (Sigma Aldrich™ Burlington, MA, USA) was added. After 10 min, 15 mL of concentrated hexane extract was added to the third flask (c) containing diluted 30 mL of methanol extract. The reactions were carried out at 65 °C for 3 h. The mixtures were then centrifuged at 10,000 rpm at 12 °C for 10 min (5810 R Eppendorf™, Hamburg, Germany) to collect the pellets. OMAgNs pellets were washed three times with ultrapure water (Milli-Q, Merck™, Burlington, MA, USA) and exposed to oven drying at 80 °C (Pol-Eko). As-synthesized methanolic (M-OMAgNs), acetone (A-OMAgNs), and methanol-hexane (MH-OMAgNs) were ground and subjected to further analysis.

### 2.3. Characterization of OMAgNs

#### 2.3.1. LC/MS Analysis

Chromatographic separation and identification of bioactive molecules were performed using the UHPLC systems (Thermo Fisher™, Waltham, MA, USA) equipped with a Kinetex 2,6u C18 (100 × 2.10 mm) and a photodiode array detector (Thermo Fisher™). The samples before 5 µL of samples were separated at 40 °C. The mobile phases used for chromatographic separation were water with 0.1% formic acid (Buffer A) and acetonitrile (Buffer B). The following gradient was used: 5% B to 1 min, 15% B to 2 min, 20% B to 9 min, 99% B to 15 min, isocratic 99% B for 1 min, 5% B to 17 min, and re-equilibration for 3 min. The flow rate was set at 0.350 mL min^−1^. The temperature of the sample manager was 8 °C.

Plant compounds were identified using two systems of liquid chromatography coupled to mass spectrometers. The first system consisted of an Impact HD (Bruker™, Billerica, MA, USA) ion trap mass spectrometer with quadrupole time-of-flight (QTOF) analyzer, and the second comprised a UHPLC UltiMate 3000 system (Dionex™, Sunnyvale, CA, USA; Thermo Fisher Scientific™). The absorption of identified molecules was measured at 254 nm wavelength. Compounds were identified according to the exact masses of their [M + H] + ions and their fragmentation patterns and chromatographic retention times by comparison with compounds previously purified and identified using NMR methods.

#### 2.3.2. Fourier Transform Infrared Spectroscopy (FT-IR)

FTIR analysis was carried out to observe the presence of vibrational modes on the surface of the prepared sample. FT-IR spectroscopy for the synthesized sample was performed using KBr pellet methodology in the range of 650–4000 cm^−1^ (Jasco 4700A, Jasco, Tokyo, Japan).

#### 2.3.3. UV Spectroscopy

UV analysis was used to detect the absorption spectra of the samples. The analysis was performed using Denovix DS-11 FX, UV-Vis mode, with an initial volume of 2 µL. The results were processed using automated path length correction.

#### 2.3.4. X-ray Diffraction (XRD)

To inspect the crystallographic configuration of green fabricated OMAgNs, XRD analysis was carried out using Oxford Diffraction four-wheel X-ray diffractometer, radiation source lamp with Mo anode, and wavelength 0.71069 Å CCD radiation detector model EOS S2. The results were recalculated for Cu anode wavelength 1.5406 Å.

#### 2.3.5. Scanning Electron Microscopy (SEM) and Energy Dispersive Spectroscopy (EDS)

OMAgNs size and nanostructure were determined by QUANTA 250 FEG, FEI scanning electron microscopy (SEM) operating between 10 and 30 kV, depending on the magnification. The samples were prepared at the stages covered with carbon tape. The energy dispersive spectroscopy (EDS) images were obtained for quantitative analysis and chemical composition mapping.

#### 2.3.6. Thermogravimetric Analysis (TGA)

Thermogravimetric analysis of all prepared samples was performed using (PerkinElmer™, Waltham, MA, USA) TGA 4000 analyzer. The powdered samples were subjected to a heating range of 30–995 °C at 10 °C/min. In the process of analysis, nitrogen gas was applied at 20.0 mL/min.

#### 2.3.7. Zeta Potential

To inspect the zeta potential of prepared NS, Nanosizer ZS Malvern™ was used, combined with Zetasizer Panalytical Malvern™ software (v7.10). The sample containing 0.1 mg was suspended in 4 mL of ddH_2_O and sonicated for 30 min in an ultrasonic bath (Sonorex Digiplus Badelin™). Zeta potential measurements were carried out by diluting the sample to a dispersed phase fraction of approximately 10^−5^.

#### 2.3.8. Dye Degradation

Dye solution (Brilliant Blue R, Sigma™) was prepared by adding 0.828 mg of dye to 10 mL of ultrapure water (Mili-Q, Merck™). Similarly, 18.57 mg of M-OMAgNs was added to 10 mL of ultrapure water (Mili-Q, Merck™). The prepared dye solution (0.828 mg/mL) was divided into six Eppendorf tubes (Eppendorf Quality™), each of 0.5 mL, and the same volume of an aqueous solution of as-synthesized M-OMAgNs (1.857 mg/mL) was impregnated in it. Immediately after adding M-OMAgNs, the vial was exposed to sunlight for the photoexcitation of M-OMAgNs. Samples were collected at 5, 10, 15, 20, and 30 min intervals. They were then centrifuged at 15,000 rpm for 10 min to remove the pellets of M-OMAgNs. The pellets were then reused for the second and third round of the same experiment. The diluted methanolic extract (1.5 mL of extract in 8.5 mL of ddH_2_O) prepared for M-OMAgNs synthesis was also used for dye degradation using the same methodology, with only one round performed. To examine the photocatalytic performance of M-OMAgNs and extract, samples (residue after centrifugation) were subjected to UV analysis.

## 3. Results

### 3.1. LC/MS

Compounds identified by the retention time (RT) and mass to charge ratio (*m/z*) are listed in Table 1. *m*/*z* parameters were obtained from the eluate analysis with the coupled mass spectrometer. There were a few notable compounds abundant in the extracts—caffeoylquinic acid, caffeic acid, 11,13-dihydro-8-deoxylactucin, and stigmasterol (Supplementary Figure S1A,C,E). From the chromatograms after synthesis (Supplementary Figure S1B,D,F), it can be assumed that all of the caffeic acid and stigmasterol were used in the synthesis reaction, because their respective peaks are no longer present.

### 3.2. FTIR

Supplementary Figure S2A–C presents FTIR spectra of the respective extracts before and after synthesis of OMAgNs. In the majority of these spectra, particular peaks can be distinguished. Peaks at 3326–3346 cm^−1^ show -OH groups attached to many of the alcohols and phenols present in the samples. The Alkyl -CH bond corresponds to a 2852–2883 cm^−1^ peak, while Amides -CO and –NH groups are represented by 1636–1641 cm^−1^ and 1401–1411 cm^−1^ regions, respectively. Absorption bands at 1486 cm^−1^ and 1341–1362 cm^−1^ are indicative of NO groups of nitro compounds. At 1267–1288 cm^−1^, 1221–36 cm^−1^, 1117 cm^−1^, and 1015–16 cm^−1^ peaks correspond to the -CO stretching vibrations of esters and ethers. In the 780–730 cm^−1^ range, deformation vibrations of the C-H bond in the aromatic rings are visible in the spectra. In acetone extract, there is a specific ketenimine C = N absorption band at 1694 cm^−1^ representing a variety of secondary metabolites. A notable difference is visible between methanol extracts before and after synthesis, suggesting that the majority of compounds were utilized in the synthesis of OMAgNs.

Supplementary Figure S2D shows the spectra of synthesized OMAgNs with various extracts. Similar to other spectra, the peak at 3436 cm^−1^ shows the -OH group present in alcohols and phenols. Alkyl -CH group corresponds to 2919 cm^−1^ and 1381 cm^−1^. The 2850 cm^−1^ band represents the C–H stretching of aromatic compounds. An abundance of ammonium ions is shown at 2353 cm^−1^ and 2321 cm^−1^, in addition to the C = N group at 1630 cm^−1^. Specific peaks are visible in the spectrum of OMAgNs prepared with methanol extract. A variety of metabolites also manifest themselves as affluence of carbonyl C = O group at 1727 cm^−1^ from aldehydes and/or in carboxylic acids at 1240.94 cm^−1^ in aromatic ethers and 1160.94 cm^−1^ in esters. -N-H stretch vibrations from amide bonds in the proteins are visible at 1451.17 cm^−1^. These functional groups have a role in the stability/capping of OMAgNs, as reported in previous studies [31]. Halogens are present at 1030.77 cm^−1^. The peaks around 830–720 cm^−1^ are vibrations of the C-H bond in the aromatic rings. These groups are present because methanol is the most polar of the solvents used.

### 3.3. Ultraviolet-Visible Spectroscopy (UV)

**Figure 1:**
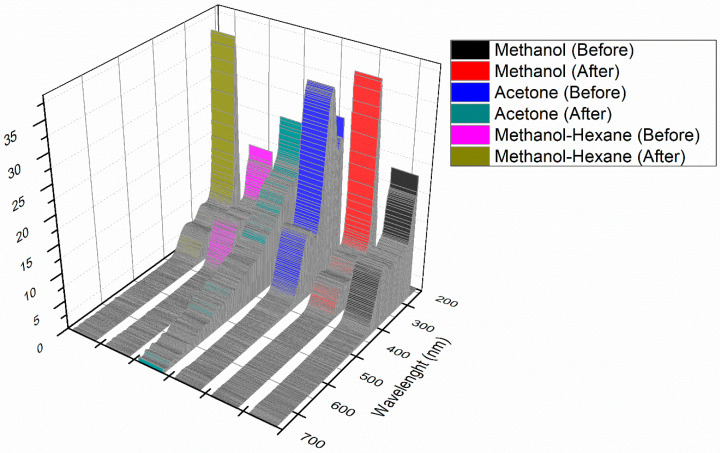
Absorption spectra of extracts before and after synthesis.

As presented in Figure 1, the presence of peaks around 250–350 nm confirms the presence of various polyphenolic compounds in the plant extract. In the case of samples taken after synthesis, there is a decrease in absorbance as compared with samples before synthesis. The results supplement previous data, indicating the utilization of plant metabolites and their functional groups in the synthesis of OMAgNs.

As shown in Supplementary Figure S3, prepared NS have slightly different absorbance values. The peaks around 220–350 nm are present due to the attachment of ligands on the NS surface. The maximum absorbance for NS is at 492 nm.

### 3.4. X-ray Diffraction Analysis (XRD)

**Figure 2:**
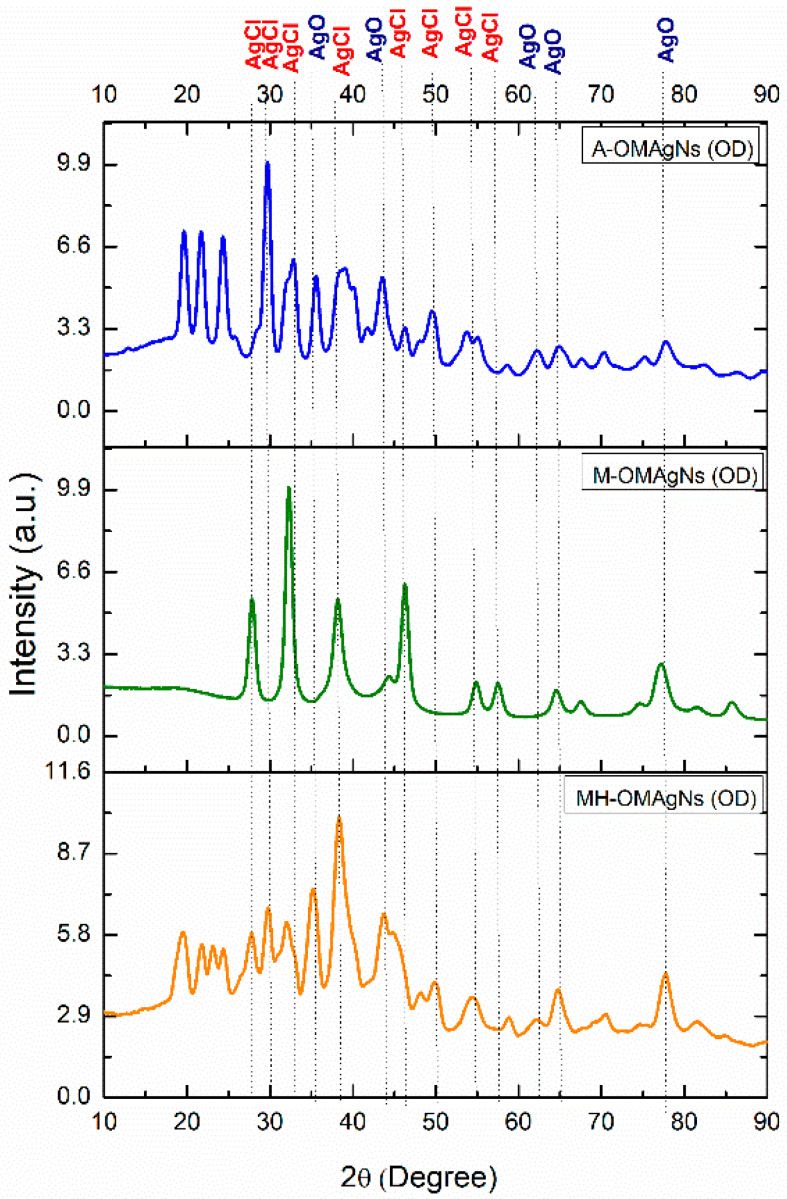
XRD spectra of synthesized OMAgNs.

To determine the crystalline planes’ growth of prepared nanostructures, an XRD analysis was performed. The results are shown in Figure 2 as a comparison between spectra of nanostructures prepared from the different extracts. The peaks around 20–25° corresponded to the presence of bioorganic matters and capping agents during OMAgNs formation.

The sample containing oven-dried A-OMAgNs showed a prominent peak at 30°, corresponding to AgCl crystalline plane. Other AgCl peaks were visible at around 32°, 38°. 50°, and 55°. The AgO crystalline peaks were present at around 36°, 62°, 65°, and 78°.

M-OMAgNs dried in the oven exhibited different growth patterns, with a dominating AgCl peak at around 32°. Interestingly, most of the crystalline planes contained AgCl with values at approximately 28°, 38°, 46°, 55°, and 57°. AgO planes were confirmed at around 65° and 78°.

The most intense peak in MH-OMAgNS was shown at around 38°, as in other samples affiliated to AgCl. There were many less intense AgCl peaks at approximately 28°, 30°, 32°, 38°, 50°, and 55°. Moreover, AgO peaks were present at 36°, 65°, and 78°.

### 3.5. SEM and EDS Analysis

As presented in Supplementary Figure S3, prepared A-OMAgNs had a spherical shape with sizes in the range of 30–50 nm. The particles showed a strong tendency to form irregular aggregates due to the ligands present on their surface.

As shown in Supplementary Figure S4, the M-OMAgNs nanoparticles were in the 20–25 nm range, forming spheres, then gathered into elongated agglomerates. The nanoparticles were visible in complexes, placed close to each other.

As shown inn Supplementary Figure S5, silver nanoparticles had a size range around 20–30 nm, forming nanowires. There were also visible parts of nanowires in the image background, probably due to high-temperature exposure. The synthesis of nanowires may have been assisted by some non-polar compounds extracted by hexane.

EDS analysis confirmed the presence of silver in all of the samples. In the case of A-OMAgNs, there was the highest signal from silver, possibly due to the highest yield and concentration of OMAgNs in the investigated sample. The high signal from carbon in the two other OMAgNs can be attributed to the carbon tape, which was used as a mounting agent.

### 3.6. Thermogravimetric Analysis (TGA)

**Figure 3:**
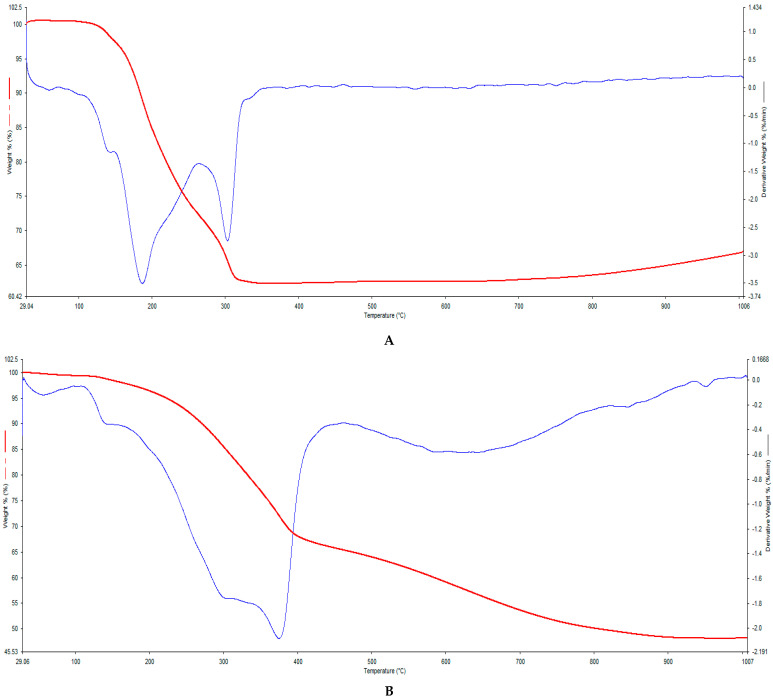

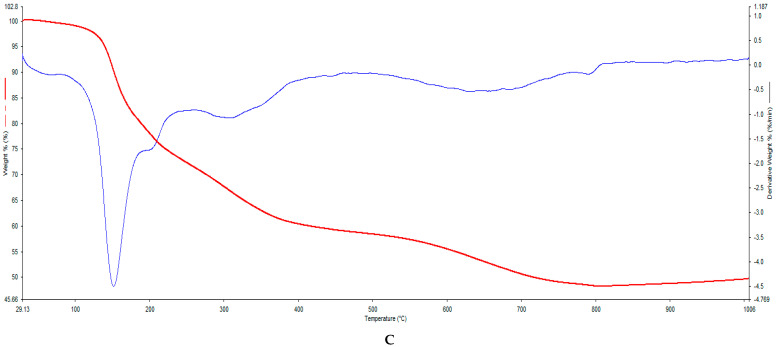
TGA curve (red) and DTG curve (blue) of A-OMAgNs (**A**), M-OMAgNs (**B**), and MH-OMAgNs (**C**).

In Figure 3A, A-OMAgNs, there were two major peaks connected with substantial mass loss. The first decrease appeared around 150–200 °C, connected to the loss of carbon from low molecular weight and thermally unstable compounds. The molecular oxygen present in their structure facilitated the formation of carbon monoxide and carbon dioxide with increasing temperature. The second decrease indicates the presence of flavonoids in the sample, which lost carbon at around 300 °C, in the form of carbon oxide and carbon dioxide. The loss of water was confirmed at around 100 °C.

As shown in Figure 3B, M-OMAgNs, there was a visible water loss at around 100 °C, followed by carbon loss in flavonoids at about 300–400 °C. Due to the structural differences resulting in distinctive oxygen composition, at 600–800 °C, high-temperature carbon and oxygen loss was present.

As shown in Figure 3C, MH-OMAgNs, the major weight decrease was present at around 150 °C, belonging to the unstable compounds carbon loss present in the sample. As in previous results, there was also visible water loss at about 100 °C and flavonoids carbon loss at 300–400 °C. The slight decrease in the 500–800 °C range indicates the high-temperature oxygen and carbon loss.

### 3.7. Zeta Potential Analysis

**Figure 4:**
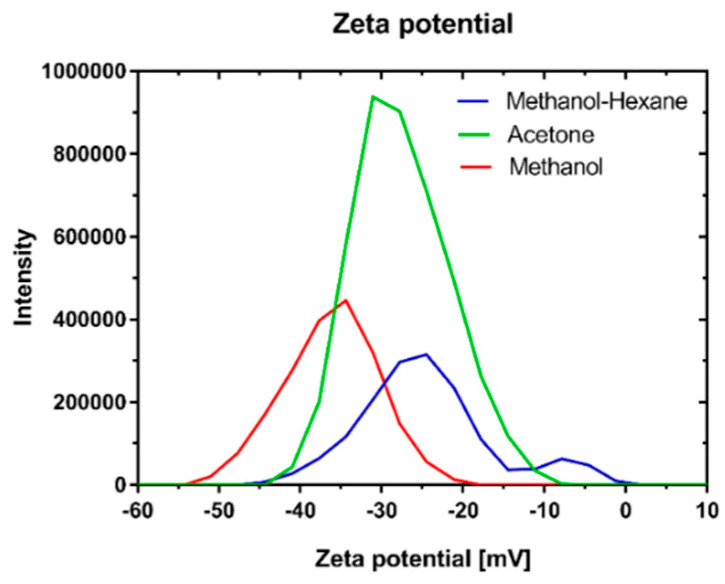
Zeta potential graphs of A-OMAgNs with a maximum value of ζ = −28.6 mV; M-OMAgNs with a maximum value of ζ = −37.3 mV; MH-OMAgNs with a maximum value of ζ = −30.7 mV.

The Zeta potentials of OMAgNs were as follows: A-OMAgNs with a value of ζ = −28.6 m, M-OMAgNs with a value of ζ = −37.3 mV, and MH-OMAgNs with a value of ζ = −30.7 mV. OMAgNs prepared with methanol extract had the highest negative value of zeta potential (Figure 4). The zeta potential value showed the relative difference in charge between the surface of the particle measured at the hydrodynamic radius of the particle and the solvent—the higher the value, the more stable it was in the solution. Since methanol was the most hydrophilic solvent from the three mixtures used in the paper, the extract contained the highest number of hydrophilic compounds. Therefore, the lowest value (meaning the highest difference in surface charge) obtained for M-OMAgNs can be attributed to the highest amount of hydrophilic and negatively charged compounds capping the surface of M-OMAgNs.

### 3.8. Dye Degradation

**Figure 5:**
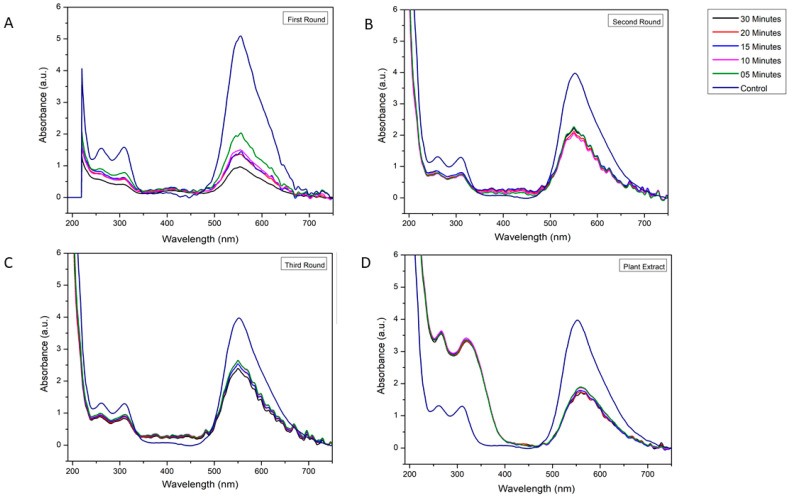
UV spectra of oven dried M-OMAgNs dye degradation in the first round (**A**), oven dried M-OMAgNs dye degradation in the second round (**B**), oven dried M-OMAgNs dye degradation in the third round (**C**), and methanol plant extract dye degradation (**D**).

**Figure 6:**
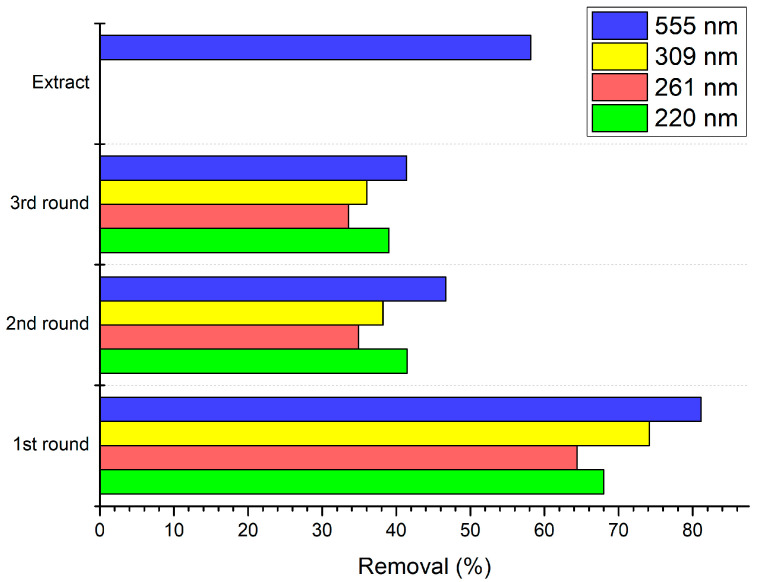
Removal of the dye after 30 min as the decrease in the absorbance values of measured peaks at 220, 261, 309, and 555 nm in the 1st, 2nd, and 3rd round of the photocatalysis, as well as degradation of the 555 nm peak by plant extract.

In Figure 5 and Supplementary Table S1, the dye photodegradation process is presented as a reduction of absorbance in a given wavelength. The control sample was the dye without added M-OMAgNs (negative control). The dye showed four peaks: at 220 nm, 261 nm, 309 nm, and 555 nm. After 5 min, absorbance was reduced by 50.3%, 45.07%, 49.62%, and 58.59%, respectively. After 10 min, further decrease was observed by 18.87%, 13.62%, 24.59%, and 29.02%, as compared to the values at the previous time intervals. There was a further reduction in 15 min by 4.82%, 4.62%, 7.82%, and 8.02%. A subsequent slight increase in the peaks at 220 nm, 261 nm, and 309 nm could be a result of conformational changes on the surface of the metalloorganic construct. After 30 min, from the beginning of the experiment, the peaks at 220 nm, 261 nm, 309 nm, and 555 nm were reduced by 67.99%, 64.41%, 74.15%, and 81.13%, respectively (Figure 6).

As shown in Figure 5B, dye photodegradation, using previously applied NS, was performed. As stated before, the dye exhibited four major peaks, and after 5 min, they decreased by 40.50%, 39.11%, 42.25%, and 45.39% in all of the examined wavelengths. After 10 min, the reaction slowed down as the absorption was further decreased by 7.8%, 2.89%, 3.89%, and 9.49%. Similar to the reaction in the first round, there was a slight increase in absorbance soon after the reduction slowed down. At the end of the second round, there was an overall decrease of 41.46%, 34.91%, 38.21%, and 46.68% (Figure 6).

As shown inn Figure 5C, the synthesized material was used again to test its photodegradation properties. The absorption was studied for the same wavelengths. After 5 min, the rapid decrease in absorbance was recorded by 30.41%, 24.98%, 26.67%, and 35.63%. However, after 10 min, the values did not exhibit major change as they decreased by 3.08%, 1.63%, and 0.74% and increased by <0.2%. Since the beginning of the experiment, after 30 min, M-OMAgNs managed to decrease absorbance values by 39.00%, 33.54%, 36.05%, and 41.37% for 220 nm, 261 nm, 309 nm, and 555 nm, respectably (Figure 6).

In Figure 5D, the dye degradation properties of methanol extract were observed. In the 200–350 nm range, there was a visible absorption increase corresponding to the functional groups of abundant polyphenolic compounds present in the extract. Interestingly, at 555 nm after 5 min, there was a decrease in absorbance by 52.74%, while after 10 min, absorbance decreased by 0.96% as compared to the previous value. In the next time intervals, there was a visible further absorbance decline. After the reaction was stopped, methanol extract managed to reduce the peak at 555 nm by 58.15%, as compared to the beginning of the experiment (Figure 6).

## 4. Discussion

### 4.1. Green Chemistry and Synthesis of Ns

*C. intybus* is a medicinally important plant known for its tonic effect upon the liver and digestive tract [32]. The documented knowledge relating to the various uses of chicory has been supported by the isolation of phytochemicals and investigations into their biological activity [33]. Previous studies showed the importance of solvents used for plant extract preparation as a tool to obtain different classes of metabolites [34]. A variety of chemical compounds has also been used during the green synthesis of OMAgNs.

**Figure 7:**
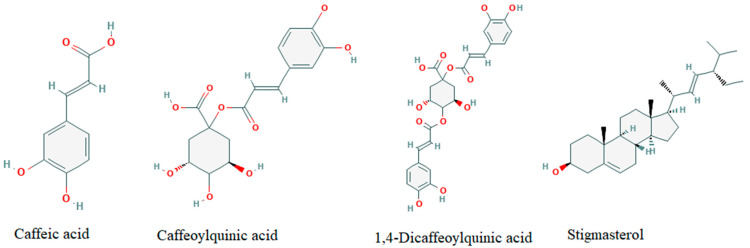
Chemical structures of reducing compounds (Source: pubchem.ncbi.nlm.nih.gov accessed on 11 August 2021).

The mixture of secondary metabolites (see Figure 7) from *C. intybus* took part in the saturation of Ag-OH complexes, followed by crystal growth from AgO to Ag^+^. In LC/MS analysis, four main compounds were identified to take part in green synthesis: caffeic acid, caffeoylquinic acid derivatives, stigmasterol, and 1,4-dicaffeoylquinic acid derivatives. (Figure S1). As reported before, caffeic acid plays an essential role in the NS synthesis, acting as a reducing agent, which allowed A-OMAgNs, M-OMAgNs, and MH-OMAgNs to obtain their small size [35]. Caffeic acid has a low molecular weight of 180.16 g/mol with high solubility of 1.16 mg/mL and a small topological polar surface area of 77.8 Å^2^, which may be the cause of its quicker activation during a reaction conducted in 65 °C [36].

The same role can be attributed to other alkaloids, caffeoylquinic acid, and 1,4-Dicaffeoylquinic acid derivatives, having a well-known antioxidant activity [37]. Moreover, 1,4-Dicaffeoylquinic acid has significant polarizability of 50.16 Å^3^, which may further facilitate NS synthesis [38].

In addition, one of the most abundant plant sterols, stigmasterol, was reported to facilitate Ag ions reduction [39], used completely during methanol-mediated and methanol-hexane-mediated NS synthesis. The presence of stigmasterol has also been detected in acetone-based extract, although its participation during the reaction is not significant. The mechanism behind A-OMAgNs synthesis may be attributed to many of the unidentified compounds present in the LC/MS chromatogram. The usage of metabolites was confirmed in UV analysis, showing an absorption decrease in the 200–350 nm wavelength. FTIR analysis also confirms the involvement of functional groups of compounds detected by changes in the intensity of the -OH group around 3000 cm^−1^. As the process continues, growing Ag atomic planes start to orient the formation of the unit cell, creating crystallite planes with different energies. Accordingly, the reaction is carried on until the well-defined growth of all Ag crystal planes.

Compounds from *C. intybus* extract were captivated to higher-energy crystallite planes, acting as capping agents to restrict further growth. Larger crystallite growth planes attracted the greatest attachment of secondary metabolites due to the large surface area-to-energy ratio at the reaction temperature of 65 °C. FTIR study show changes in the 1500–1000 cm^−1^, corresponding to -CO, -NH, and -NO groups, which may indicate the metabolites attachment. The reaction would continue until all activated capping agents react with the compounds present in the reaction mixture. As a result, the cluster of crystallite aggregates defines the shape of OMAgNs.

### 4.2. Chemical, Optical, and Crystallographic Study of OMAgNs

The FTIR spectra of OMAgNs show the clear presence of -CO, -C = N, -C = C, -C-H groups forming metalorganic constructs on their surfaces. The peaks corresponding to the -N-H and -N = O vibrations, representing functional groups of proteins and metabolites, are bound to the silver core of prepared NS, as reported in the green chemistry of Ag NS [40]. Additionally, various concentrations of nitro compounds during the reaction can affect agglomeration, as reported before [41]. The prepared A-OMAgNs, M-OMAgNs, and MH-OMAgNs showed their maximum absorption peak at 492 nm, which is in agreement with previously reported studies [42].

The crystallographic study of OMAgNs showed peaks belonging to AgO due to the presence of oxygen and AgCl due to the abundance of Cl^−^ ions in the plant extract. Chlorine (Cl^−^) ions are abundantly present in plants and play a vital role during photosynthesis and maintaining overall homeostasis [43]. The formation AgCl crystal phases in plant extract mediated synthesis could be due to the participation of electronegative Cl^−^ ions coming from the plant extract. During the secondary metabolites mediated metal (Ag^+^) ions reduction, Ag^+^ forms complex Cl^−^ ions to achieve stability, which probably resulted in the formation of the AgCl crystalline phase in the overall structure of OMAgNs. Resulting analyses show the various intensity of peaks contributing to different symmetry, proving crystallographic defects in the prepared nanostructures (defect chemistry). The peaks of Ag NS have been reported before [44,45,46,47,48]. There is also a confirmed presence of bioorganic matters and capping agents during OMAgNs formation at around 20–25°, not only present in the case of M-OMAgNs. The difference in the dominant peaks of OMAgNs shows a change in their crystallite growth pattern, contributing to variation in structure.

### 4.3. Morphological, Elemental, and Thermal Examination of OMAgNs

The size and shape of the prepared OMAgNS were determined by using SEM. The obtained size varies from around 15 to 30 nm, creating agglomerates up to 50 nm. The structures are spherical or spherical-like, exhibiting heterogeneous morphology, with some of them forming elongated structures or nanowires. EDS analysis used to determine the chemical composition of the sample shows the most prominent silver peak in the A-OMAgNs sample, with the lowest peak corresponding to the carbon. The increased value of carbon peak in all the other samples reveals the significance of the metalorganic construct on the surface of NS.

TGA analysis allowed the determination of the thermal stability of the samples. The results revealed the presence of water, more abundant in the room-temperature-dried M-OMAgNs. Flavonoid content loss was confirmed at the mass loss around 300 °C, with some of the low molecular compounds loss around 30–300 °C. Only in the M-OMAgNs and MH-OMAgNs samples, there was a visible oxygen loss at 600–1000 °C, indicating increased availability of molecular oxygen in the samples.

Zeta potential study was used to determine the stability of prepared NS in colloidal dispersions. The least stable was A-OMAgNs, with ζ = −27,6 mV, while the more stable were MH-OMAgNs, with the value of ζ = −28.6 mV, and the most stable was the M-OMAgNs, with ζ = −37.3 mV. Obtained MH-OMAgNs were prepared with the addition of hexane; therefore, more nonpolar molecules were present in the extract, which lowered the charge on the surface on MH-OMAgNs compared to M-OMAgNs and therefore diminished the stable dispersion in water. In comparison, M-OMAgNs were rich in polar compounds and easily dispersed in water; therefore, they had a high zeta potential value.

### 4.4. Dye Degradation

**Figure 8:**
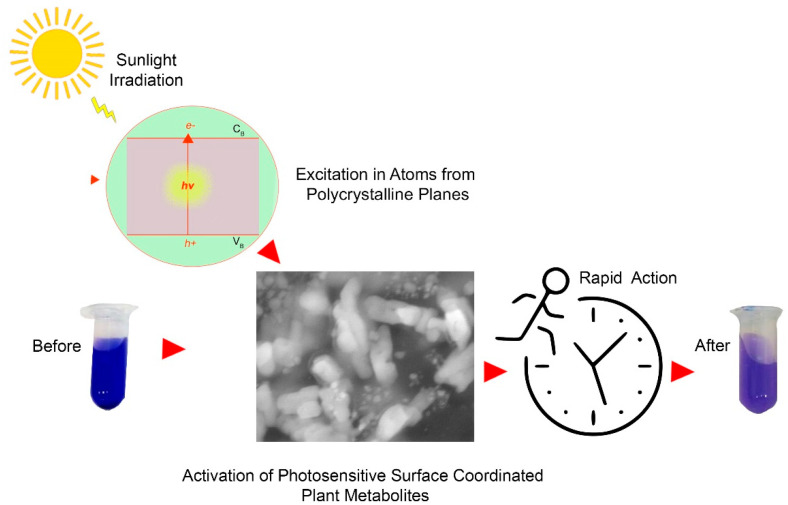
Hypothetical explanation of the photocatalytic experiment.

M-OMAgNs, due to their high yield and high polarity of used extract, resulting in high zeta potential value, were chosen as nanostructures with the most prominent catalytic activity in water. The degradation of Brilliant Blue R dye took place in the presence of sunlight. The proposed mechanism of this action is depicted in Figure 8.

Upon light irradiation, the electrons in the valence band absorb energy; hence they are promoted to the conduction band, creating holes in the conduction band. Developed charge carriers separate from each other and migrate, reaching the active sites on the surface to catalyze succeeding reactions [49,50]. The metalorganic construct was proposed to have the role of semiconductor, further facilitating the process [51].

To study the kinetics of the performed reaction, the Langmuir–Hinshelwood model was applied, using the below equation:

$$\ln C_{0}/C_{f}=k*t$$

where *C*_0_ represents initial dye concentration, *C_f_* is the final dye concentration, *t* corresponds to the contact time, and *k* is the photocatalytic rate constant. Initial and final dye concentration was measured from the UV spectrum using absorbance value. The degradation rate using M-OMAgNs in the first round was calculated as 0.0556 min^−1^, the second round as 0.0209 min^−1^, and the third round as 0.0178 cm^−1^, while the extract had a 0.0407 min^−1^ degradation rate. The catalytic performance of extract without NS signifies the importance of organic compounds taking part in the photocatalytic reaction. However, when mixed with synthesized OMAgNs, the degradation rate almost doubled, showing a significant decrease with their second use. When OMAgNs were applied for the third time, the degradation rate showed a minor decline. The results show the synergistic effect of organic compounds from the extract acting together with prepared Ag NS to efficiently degrade Brilliant Blue R dye.

## 5. Conclusions

In conclusion, we prepared three different extracts from the *C. intybus* plant and used them to synthesize organometallic silvern nanoparticles (OMAgNs). From the three extracts, based on their high yield, physico-chemical properties, and cost efficiency, OMAgNs prepared with methanolic extract (M-OMAgNs) were used to degrade Brilliant Blue R using only sunlight irradiation and confirmed their photocatalytic activity, reducing dye absorption rapidly by half in less than 5 min.

## Figures and Tables

**Table 1:** Table with identification of compounds present in the extracts using LC/MS.

Compound	RT	*m*/*z*
Breakdown of 1,4-dicaffeoylquinic acid	0.9	203
Caffeoylquinic acid	3.4	179
Caffeic acid	4.6	179
Linoleic acid	5.7	277
11,13-dihydro-8-deoxylactucin	9.1	261
stigmasterol	13.4	411

## Supplementary Materials

The following are available online at https://www.mdpi.com/article/10.3390/biology10080784/s1, Figure S1: Chromatograms obtained for A-OMAg-Ns before (A) and after (B) synthesis, M-OMAgNs before (C) and after (D) synthesis, MH-OMAgNs before (E) and after (F) synthesis. Figure S2: FTIR spectra before and after synthesis of: acetone (A), methanol (B), methanol-hexane (C) and synthesized OMAgNs (D). Figure S3: Absorption spectra of synthesized OMAgNs. Figure S4: SEM images at 100,000× (A), 300,000× (B) magnification and EDX (C) analysis of A-OMAgNs. Figure S5: SEM images at 100,000× (A), 300,000× (B) magnification and EDX (C) analysis of M-OMAgNs. Figure S6: SEM images at 100,000× (A), 300,000× (B) and EDX (C) analysis of MH-OMAgNs. Table S1: Absorbance values for first, second and third round of M-OMAgNs and extract (shown as a.u.).

## References

[1] Marzouk Trifi I., Trifi B., Ben Souissi E., Hamrouni B. (2020). Response surface methodology for dyes removal by adsorption onto alginate calcium. Environ. Technol..

[2] Tara N., Siddiqui S.I., Rathi G., Chaudhry S.A., Inamuddin, Asiri A.M. (2019). Nano-engineered Adsorbent for the Removal of Dyes from Water: A Review. Curr. Anal. Chem..

[3] Crini G., Lichtfouse E. (2019). Advantages and disadvantages of techniques used for wastewater treatment. Environ. Chem. Lett..

[4] Jacob J.A., Biswas N., Mukherjee T., Kapoor S. (2011). Effect of plant-based phenol derivatives on the formation of Cu and Ag nanoparticles. Colloids Surf. B Biointerfaces.

[5] Raveendran P., Fu J., Wallen S.L. (2003). Completely “Green” Synthesis and Stabilization of Metal Nanoparticles. J. Am. Chem. Soc..

[6] Begum N.A., Mondal S., Basu S., Laskar R.A., Mandal D. (2009). Biogenic synthesis of Au and Ag nanoparticles using aqueous solutions of Black Tea leaf extracts. Colloids Surf. B Biointerfaces.

[7] Mosaviniya M., Kikhavani T., Tanzifi M., Tavakkoli Yaraki M., Tajbakhsh P., Lajevardi A. (2019). Facile green synthesis of silver nanoparticles using Crocus Haussknechtii Bois bulb extract: Catalytic activity and antibacterial properties. Colloids Interface Sci. Commun..

[8] Jyoti K., Baunthiyal M., Singh A. (2016). Characterization of silver nanoparticles synthesized using Urtica dioica Linn. leaves and their synergistic effects with antibiotics. J. Radiat. Res. Appl. Sci..

[9] Iqbal J., Abbasi B.A., Mahmood T., Kanwal S., Ahmad R., Ashraf M. (2019). Plant-extract mediated green approach for the synthesis of ZnONPs: Characterization and evaluation of cytotoxic, antimicrobial and antioxidant potentials. J. Mol. Struct..

[10] Ratan Z.A., Haidere M.F., Nurunnabi M., Shahriar S.M., Ahammad A.J.S., Shim Y.Y., Reaney M.J.T., Cho J.Y. (2020). Green Chemistry Synthesis of Silver Nanoparticles and Their Potential Anticancer Effects. Cancers.

[11] Logeswari P., Silambarasan S., Abraham J. (2013). Ecofriendly synthesis of silver nanoparticles from commercially available plant powders and their antibacterial properties. Sci. Iran..

[12] Gopinath V., MubarakAli D., Priyadarshini S., Priyadharsshini N.M., Thajuddin N., Velusamy P. (2012). Biosynthesis of silver nanoparticles from Tribulus terrestris and its antimicrobial activity: A novel biological approach. Colloids Surf. B Biointerfaces.

[13] Kumar D.A., Palanichamy V., Roopan S.M. (2014). Green synthesis of silver nanoparticles using Alternanthera dentata leaf extract at room temperature and their antimicrobial activity. Spectrochim. Acta Part A Mol. Biomol. Spectrosc..

[14] Nakkala J.R., Mata R., Gupta A.K., Sadras S.R. (2014). Biological activities of green silver nanoparticles synthesized with Acorous calamus rhizome extract. Eur. J. Med. Chem..

[15] Manimegalai G., Shantha Kumar S., Sharma C. (2011). Pesticide mineralization in water using silver nanoparticles. Int. J. Chem. Sci..

[16] Fouda M.M.G., Abdelsalam N.R., El-Naggar M.E., Zaitoun A.F., Salim B.M.A., Bin-Jumah M., Allam A.A., Abo-Marzoka S.A., Kandil E.E. (2020). Impact of high throughput green synthesized silver nanoparticles on agronomic traits of onion. Int. J. Biol. Macromol..

[17] Gallucci M.N., Fraire J.C., Ferreyra Maillard A.P.V., Páez P.L., Aiassa Martínez I.M., Pannunzio Miner E.V., Coronado E.A., Dalmasso P.R. (2017). Silver nanoparticles from leafy green extract of Belgian endive (*Cichorium intybus* L. var. *sativus*): Biosynthesis, characterization, and antibacterial activity. Mater. Lett..

[18] Behboodi S., Baghbani-Arani F., Abdalan S., Sadat Shandiz S.A. (2019). Green Engineered Biomolecule-Capped Silver Nanoparticles Fabricated from *Cichorium intybus* Extract: In Vitro Assessment on Apoptosis Properties Toward Human Breast Cancer (MCF-7) Cells. Biol. Trace Elem. Res..

[19] Mahmoodzadeh H., Nasr N., Saeedian E. (2016). Seed Germination and Growth Response of Chicory (*Cichorium intybus* L.) to Copper Oxide Nanoparticles. Jordan J. Agric. Sci..

[20] Torabi N., Nowrouzi A., Ahadi A., Vardasbi S., Etesami B. (2019). Green synthesis of gold nanoclusters using seed aqueous extract of *Cichorium intybus* L. and their characterization. SN Appl. Sci..

[21] Brunelle J.L., Green R. (2014). Coomassie blue staining. Methods in Enzymology.

[22] Makino A., Mae T., Ohira K. (1911). Agricultural and Biological Chemistry Colorimetric Measurement of Protein Stained with Coomassie Brilliant Blue R on Sodium Dodecyl Sulfate-Polyacrylamide Gel Electrophoresis by Eluting with Formamide Colorimetric Measurement of Protein Stained with Cooma. Agric. Biol. Chem..

[23] Kurien B.T., Scofield R.H. (2012). Accelerated coomassie blue staining and destaining of SDS-PAGE gels with application of heat. Methods Mol. Biol..

[24] Kavet R., Nauss K.M. (1990). The toxicity of inhaled methanol vapors. Crit. Rev. Toxicol..

[25] Rajan K.G., Davies B.H. (1989). Reversible airways obstruction and interstitial pneumonitis due to acetic acid. Br. J. Ind. Med..

[26] Lauby-Secretan B. (2009). Exposure to Hazardous Substances in a Standard Molecular Biology Laboratory Environment: Evaluation of Exposures in IARC Laboratories. Ann. Occup. Hyg..

[27] Shanker U., Rani M., Jassal V. (2017). Degradation of hazardous organic dyes in water by nanomaterials. Environ. Chem. Lett..

[28] Khan M.A., Alam M.M., Naushad M., Alothman Z.A., Kumar M., Ahamad T. (2015). Sol-gel assisted synthesis of porous nano-crystalline CoFe_2_O_4_ composite and its application in the removal of brilliant blue-R from aqueous phase: An ecofriendly and economical approach. Chem. Eng. J..

[29] Khan Z.U.H., Khan A., Shah A., Wan P., Chen Y., Khan G.M., Khan A.U., Tahir K., Muhammad N., Khan H.U. (2016). Enhanced photocatalytic and electrocatalytic applications of green synthesized silver nanoparticles. J. Mol. Liq..

[30] Bhagat M., Anand R., Datt R., Gupta V., Arya S. (2019). Green Synthesis of Silver Nanoparticles Using Aqueous Extract of Rosa brunonii Lindl and Their Morphological, Biological and Photocatalytic Characterizations. J. Inorg. Organomet. Polym. Mater..

[31] Niraimathi K.L., Sudha V., Lavanya R., Brindha P. (2013). Biosynthesis of silver nanoparticles using *Alternanthera sessilis* (Linn.) extract and their antimicrobial, antioxidant activities. Colloids Surf. B Biointerfaces.

[32] Judžentienė A., Būdienė J. (2008). Volatile constituents from aerial parts and roots of *Cichorium intybus* L. (chicory) grown in Lithuania. Chemija.

[33] Ramakrishnamurthy S., Singaravelu G., Devadasan V., Prakasarao A. (2020). In-vitro and In-silico analysisof anti-diabetic and anti-microbial activity of *Cichorium intybus* leaf extracts. Curr. Comput. Aided. Drug Des..

[34] Dhawan D., Gupta J. (2016). Comparison of Different Solvents for Phytochemical Extraction Potential from Datura metel Plant Leaves. Int. J. Biol. Chem..

[35] Kim H.S., Seo Y.S., Kim K., Han J.W., Park Y., Cho S. (2016). Concentration Effect of Reducing Agents on Green Synthesis of Gold Nanoparticles: Size, Morphology, and Growth Mechanism. Nanoscale Res. Lett..

[36] Caffeic Acid|C9H8O4—PubChem. https://pubchem.ncbi.nlm.nih.gov/compound/Caffeic-acid.

[37] Pantoja Pulido K.D., Colmenares Dulcey A.J., Isaza Martínez J.H. (2017). New caffeic acid derivative from Tithonia diversifolia (Hemsl.) A. Gray butanolic extract and its antioxidant activity. Food Chem. Toxicol..

[38] 1,4-Di-O-caffeoylquinic Acid (FDB013317)—FooDB. https://foodb.ca/compounds/FDB013317.

[39] Kanagamani K., Muthukrishnan P., Ilayaraja M., Shankar K., Kathiresan A. (2018). Synthesis, Characterisation and DFT Studies of Stigmasterol Mediated Silver Nanoparticles and Their Anticancer Activity. J. Inorg. Organomet. Polym. Mater..

[40] Pawar O., Deshpande N., Dagade S., Waghmode S., Nigam Joshi P. (2016). Green synthesis of silver nanoparticles from purple acid phosphatase apoenzyme isolated from a new source Limonia acidissima. J. Exp. Nanosci..

[41] Ravindran A., Singh A., Raichur A.M., Chandrasekaran N., Mukherjee A. (2010). Studies on interaction of colloidal Ag nanoparticles with Bovine Serum Albumin (BSA). Colloids Surf. B Biointerfaces.

[42] Balachandar R., Gurumoorthy P., Karmegam N., Barabadi H., Subbaiya R., Anand K., Boomi P., Saravanan M. (2019). Plant-Mediated Synthesis, Characterization and Bactericidal Potential of Emerging Silver Nanoparticles Using Stem Extract of Phyllanthus pinnatus: A Recent Advance in Phytonanotechnology. J. Clust. Sci..

[43] Chen W., He Z.L., Yang X.E., Mishra S., Stoffella P.J. (2010). Chlorine nutrition of higher plants: Progress and perspectives. J. Plant Nutr..

[44] Li D., Han T., Zhang L., Zhang H., Chen H. (2017). Flexible transparent electrodes based on silver nanowires synthesized via a simple method. R. Soc. Open Sci..

[45] Han C., Ge L., Chen C., Li Y., Zhao Z., Xiao X., Li Z., Zhang J. (2014). Site-selected synthesis of novel Ag@AgCl nanoframes with efficient visible light induced photocatalytic activity. J. Mater. Chem. A.

[46] Zhao X., Zhang J., Wang B., Zada A., Humayun M. (2015). Biochemical Synthesis of Ag/AgCl Nanoparticles for Visible-Light-Driven Photocatalytic Removal of Colored Dyes. Materials.

[47] Hamed S.M., Mostafa A.M.A., Abdel-Raouf N., Ibraheem I.B.M. (2016). Biosynthesis of silver and silver chloride nanoparticles by Parachlorella kessleri SAG 211-11 and evaluation of its nematicidal potential against the root-knot nematode; Meloidogyne incognita. Aust. J. Basic Appl. Sci..

[48] Okaiyeto K., Ojemaye M.O., Hoppe H., Mabinya L.V., Okoh A.I. (2019). Phytofabrication of Silver/Silver Chloride Nanoparticles Using Aqueous Leaf Extract of Oedera genistifolia: Characterization and Antibacterial Potential. Molecules.

[49] Gautam S., Agrawal H., Thakur M., Akbari A., Sharda H., Kaur R., Amini M. (2020). Metal oxides and Metal Organic Frameworks for the Photocatalytic Degradation: A Review. J. Environ. Chem. Eng..

[50] Alkhatib I.I., Garlisi C., Pagliaro M., Al-Ali K., Palmisano G. (2020). Metal-organic frameworks for photocatalytic CO_2_ reduction under visible radiation: A review of strategies and applications. Catal. Today.

[51] Li L., Yu X., Xu L., Zhao Y. (2020). Fabrication of a novel type visible-light-driven heterojunction photocatalyst: Metal-porphyrinic metal organic framework coupled with PW12/TiO_2_. Chem. Eng. J..

