# Supplementary material for: Photodegradation of Biohazardous Dye Brilliant Blue R Using Organometallic Silver Nanoparticles Synthesized through a Green Chemistry Method
Source: Biology (Basel). 2021 Aug 17;10(8):784. doi: 10.3390/biology10080784 (PMC8389564; doi:10.3390/biology10080784)
Supplement: Supplementary file 1 [file biology-10-00784-s001.zip › biology-1327485-supplementary.pdf]

Supplementary Data

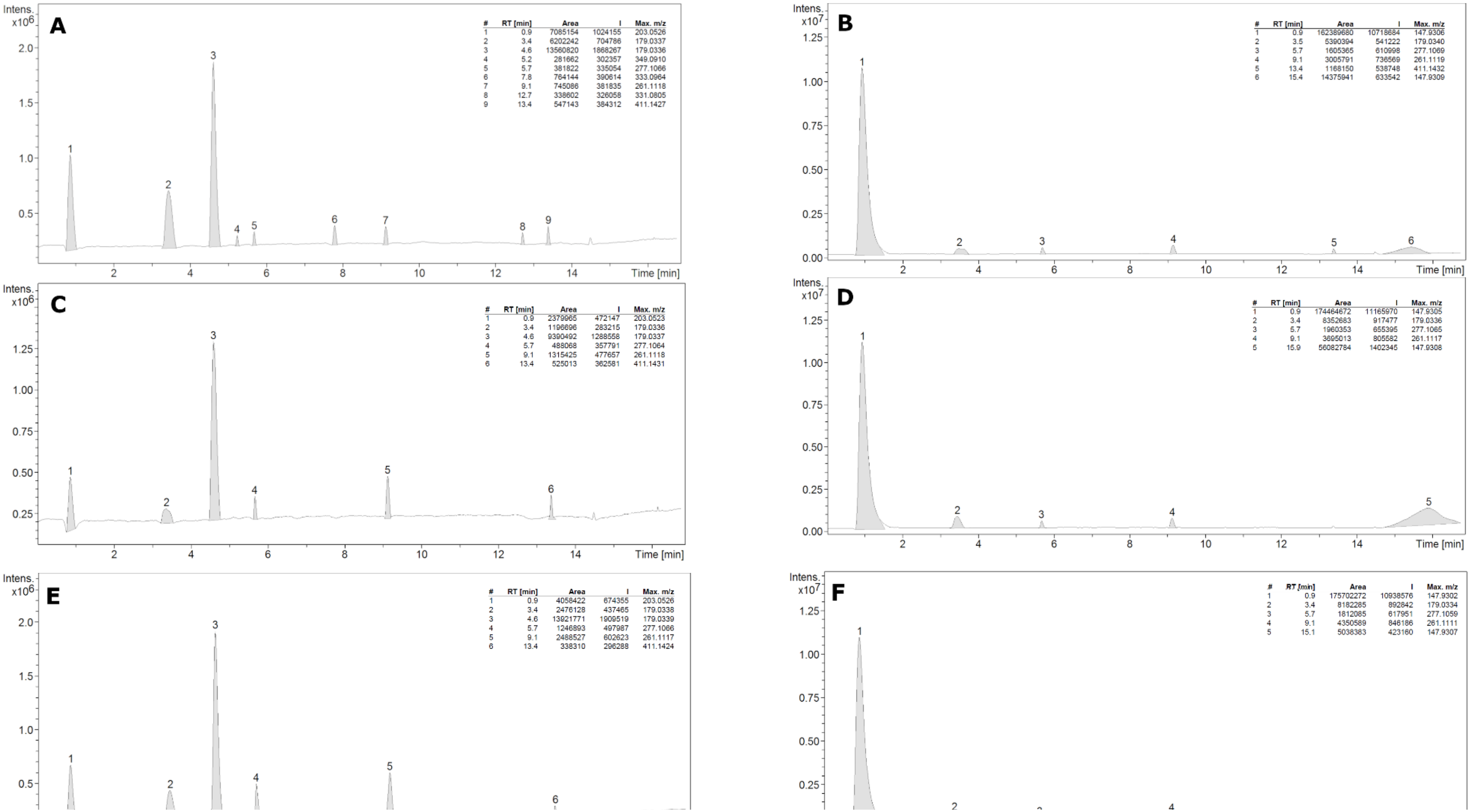

Figure S1: Chromatograms obtained for A-OMAg-Ns before (A) and after (B) synthesis, M-OMAgNs before (C) and after (D) synthesis, MH-OMAgNs before (E) and after (F) synthesis.

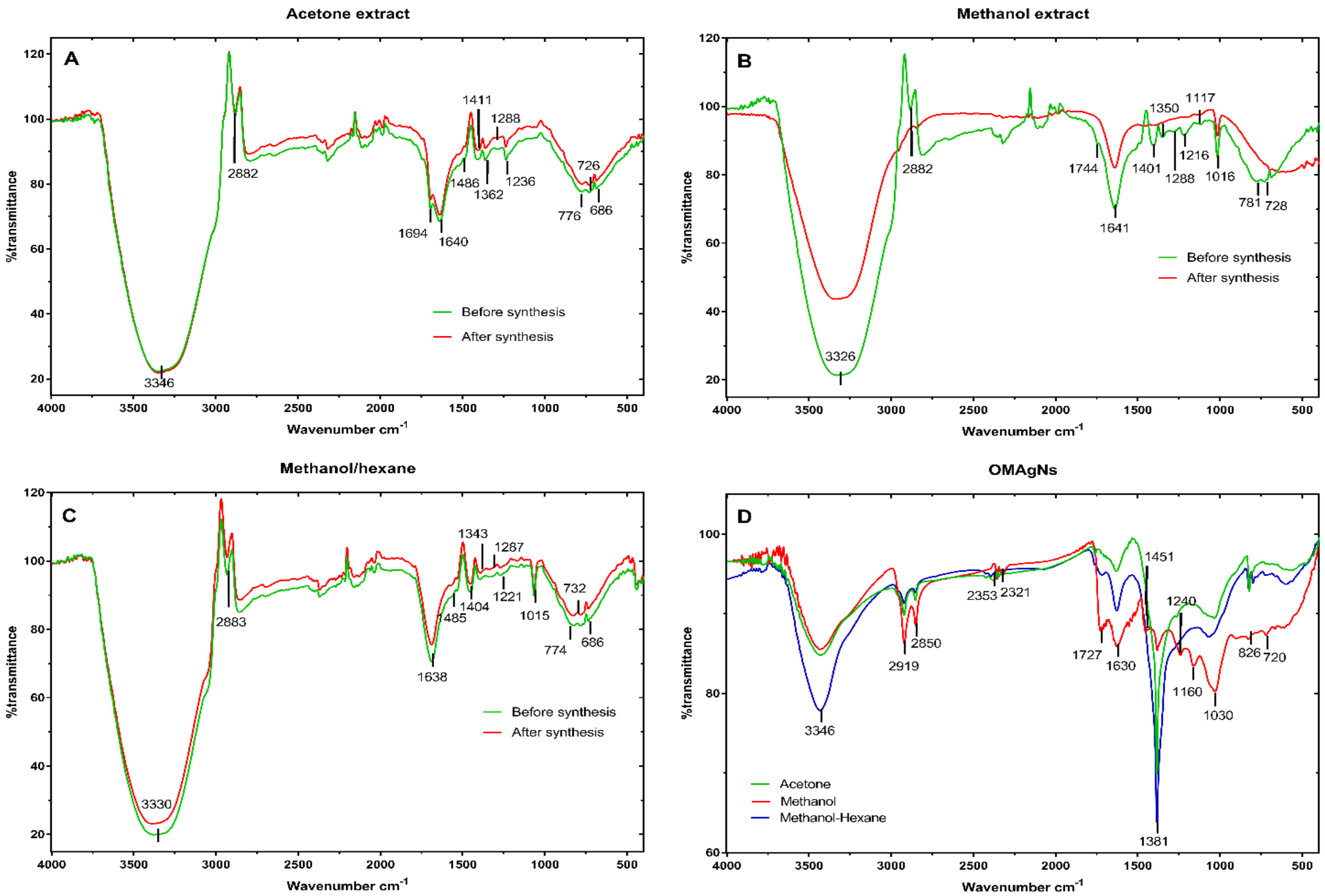

Figure S2: FTIR spectra before and after synthesis of: acetone (A),methanol (B), methanol-hexane (C) and synthesized OMAgNs (D*)*

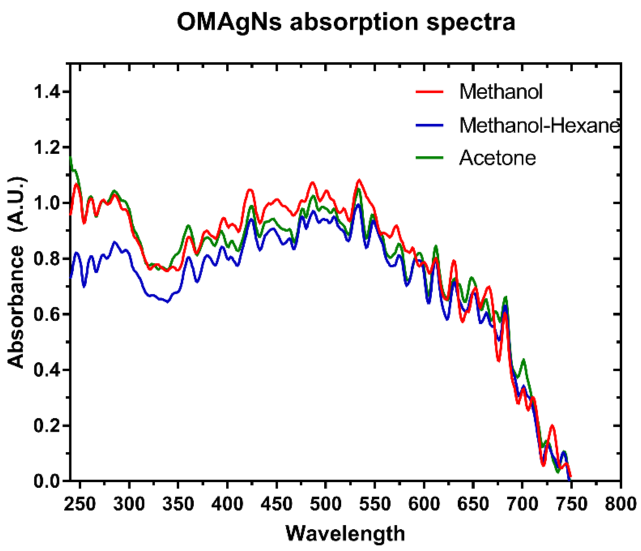

Figure S3: Absorption spectra of synthesized OMAgNs

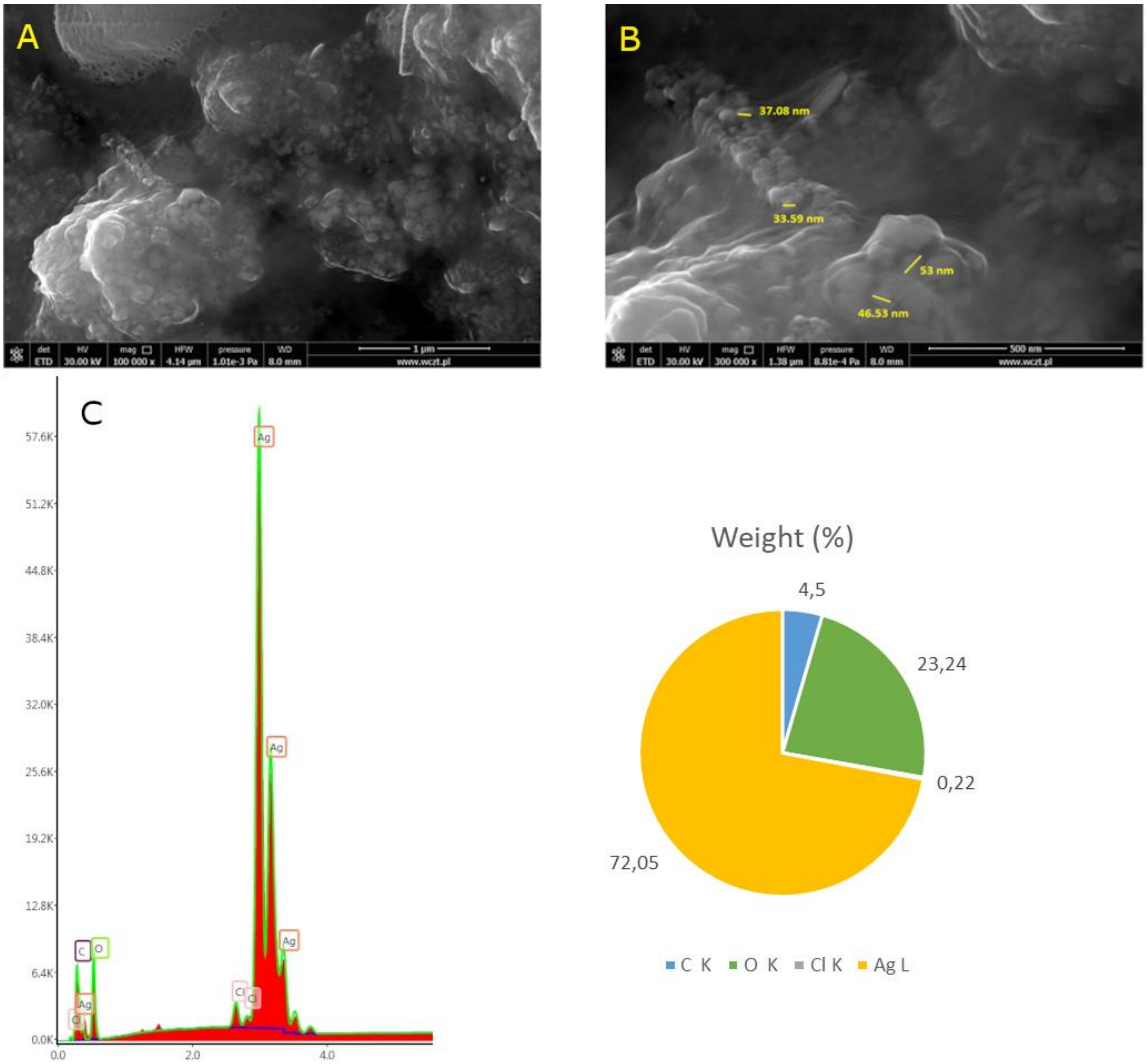

Figure S4*:* SEM images at 100000x (A), 300000x (B) magnification and EDX (C) analysis of A-OMAgNs

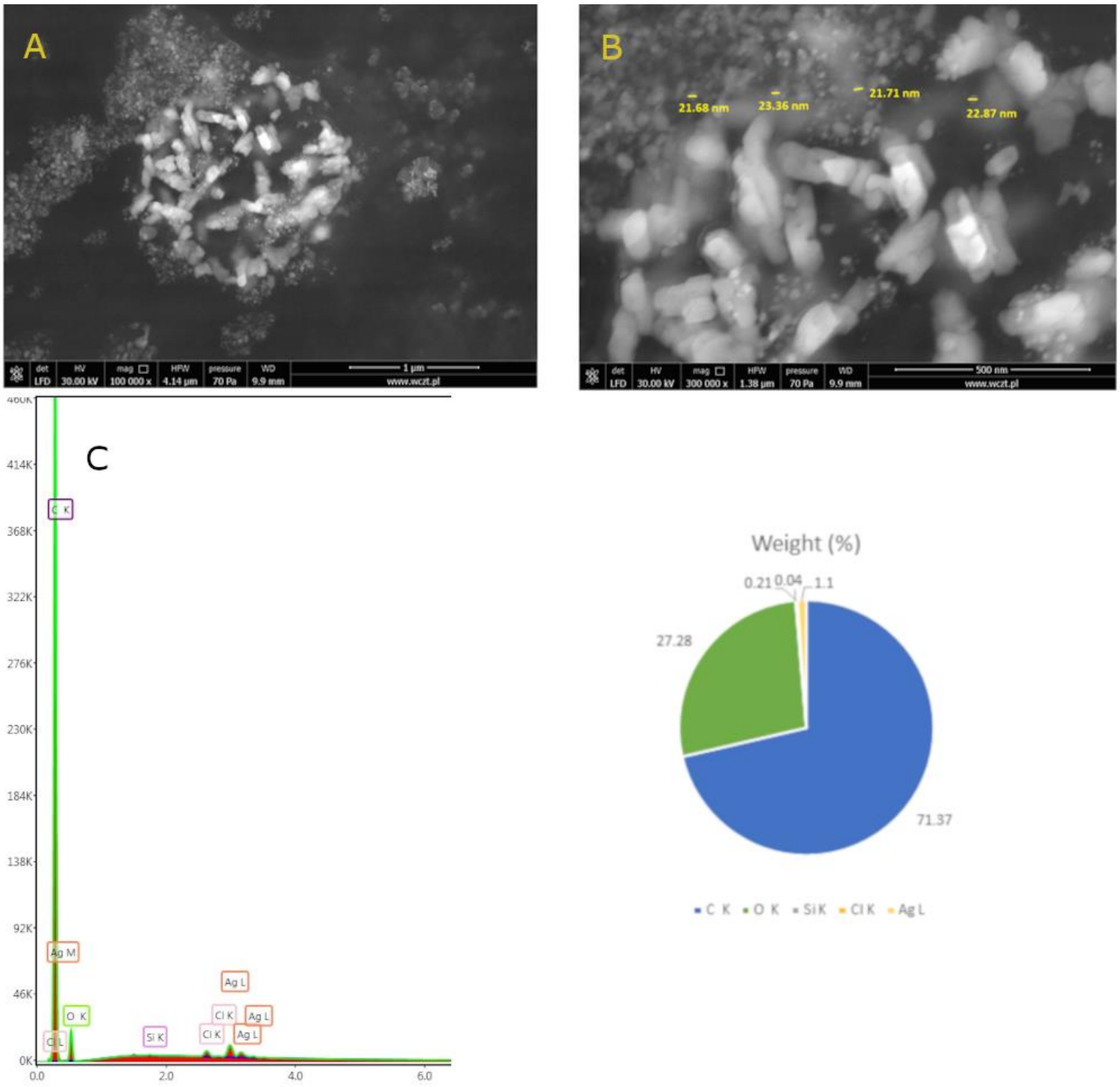

Figure S5: SEM images at 100000x (A), 300000x (B) magnification and EDX (C) analysis of M-OMAgNs

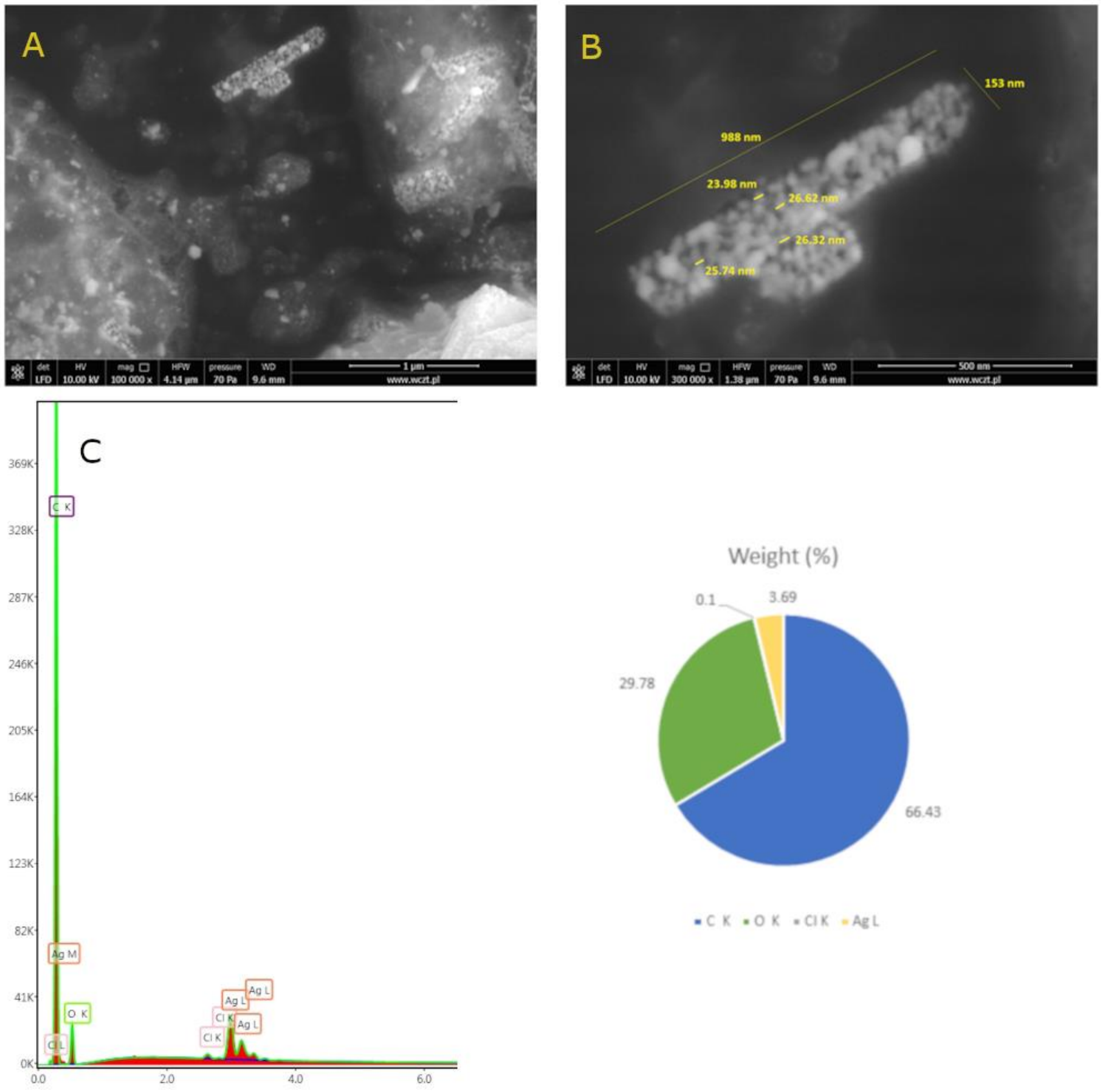

Figure S6: SEM images at 100000x (A), 300000x (B) and EDX (C) analysis of MH-OMAgNs

•

• **Table S1:** Absorbance values for first, second and third round of M-OMAgNs and extract (shown as a.u.)

	Time (min)	220 nm	261 nm	309 nm	555 nm
**1st round**	0	4.062	1.551	1.582	5.093
	5	2.019	0.852	0.797	2.109
	10	1.638	0.736	0.601	1.497
	15	1.559	0.702	0.554	1.377

	20	1.644	0.746	0.576	1.365
	30	1.300	0.552	0.409	0.961
**2nd round**	0	3.449	1.309	1.290	3.955
	5	2.052	0.797	0.745	2.160
	10	1.892	0.774	0.716	1.955
	15	2.036	0.856	0.804	2.140
	20	1.896	0.791	0.750	2.012
	30	2.019	0.852	0.797	2.109
**3rd round**	0	3.449	1.309	1.290	3.955
	5	2.400	0.982	0.946	2.546
	10	2.326	0.966	0.939	2.551
	15	2.236	0.926	0.896	2.446
	20	2.174	0.901	0.874	2.450
	30	2.104	0.870	0.825	2.319
**Extract**	0	3.449	1.309	1.290	3.955
	5	7.753	3.490	3.203	1.869
	10	7.987	3.553	3.258	1.851
	15	7.814	3.527	3.210	1.777
	20	7.703	3.498	3.156	1.727
	30	7.659	3.477	3.140	1.654
